# A case of metachronous double primary neuroendocrine cancer in pancreas/ileum and uterine cervix

**DOI:** 10.3109/03009734.2012.707254

**Published:** 2012-10-30

**Authors:** Giovanni Sisti, Anna Maria Buccoliero, Luca Novelli, Maddalena Sansovini, Stefano Severi, Annalisa Pieralli, Lorenzo Livi, Massimiliano Fambrini

**Affiliations:** ^1^Department of Science for Woman and Child Health, University of Florence, Florence, Italy; ^2^Department of Human Pathology and Oncology, University of Florence, Florence, Italy; ^3^Department of Radiometabolic Medicine, Istituto Scientifico Romagnolo per lo Studio e la Cura dei Tumori (IRST), Meldola, Italy; ^4^Department of Radiation-Oncology, University of Florence, Florence, Italy

**Keywords:** Metachronous double primary cancer, neuroendocrine cancer, pap smear, pancreas, uterine cervix

## Abstract

We describe an unusual case of a 50-year-old female patient developing two primary cancers with neuroendocrine features. Initially the patient underwent surgery for an entero-pancreatic neuroendocrine carcinoma. During the subsequent follow-up she experienced some episodes of vaginal bleeding with negative PET scanning with the tracer fluorine-18 (F-18) fluorodeoxyglucose (FDG). A Papanicolaou (pap) smear and an endometrial biopsy revealed a primary neuroendocrine cancer of the uterine cervix. The present case underlines the importance of clinical follow-up after a diagnosis of intestinal neuroendocrine tumor, investigating any new symptom. Female patients, after the diagnosis of entero-pancreatic neuroendocrine carcinoma, must be recommended to continue screening pap test examinations for the likelihood of classical squamous and glandular cervical cancers and also for neuroendocrine cervical cancer.

## Introduction

Neuroendocrine tumors (NETs) comprehend a genetically diverse spectrum of malignant solid tumors arising from the secretory cells of the neuroendocrine cell system. They occur in the gastro-intestinal tract (GIT), in the lungs, and other organs (1–4).

According to the most recent international accepted classification (5), NETs can be distinguished in well-differentiated neuroendocrine tumors, well-differentiated (low-grade) neuroendocrine carcinomas, and poorly differentiated (high-grade) neuroendocrine carcinomas (large-cell neuroendocrine and small-cell carcinomas). Small-cell carcinomas comprehend small-cell lung cancer (SCLC) and extra-pulmonary small-cell carcinomas (ESCC).

We report an unusual case of a 50-years-old woman, initially treated for a neuroendocrine cell carcinoma of both pancreas and ileum, developing another primary neuroendocrine malignancy arising from the cervix 1 year later.

According to the World Health Organization (WHO) classification (3), this patient developed a well-differentiated neuroendocrine carcinoma of both pancreas and ileum (gastro-entero-pancreatic neuroendocrine tumor (GEP-NET)) and a poorly differentiated small-cell neuroendocrine cervical carcinoma (SCNCC).

## Case presentation

A 50-year-old Caucasian woman with no previous significant diseases was admitted to the surgical department of our institution in 2010 for carcinoid syndrome.

Biochemical serum and urinary markers of GEP-NETs were assessed. Serum chromogranin A (CgA), serum neuron-specific enolase (NSE), serum serotonin (5-hydroxytryptamine) and its major product of degradation, urine 5-hydroxyindoleacetic acid (5-HIAA), revealed increased levels all over. In contrast, serum basal levels of somatostatin, gastrin, glucagon, insulin, vasoactive intestinal peptide (VIP), and pancreatic polypeptide (PP) were within their respective normal ranges.

The first staging and diagnostic imaging procedure was a somatostatin receptor scintigraphy (SRS) (Octreoscan) demonstrating the presence of a primary entero-pancreatic cancer and three metastatic sites in the interventricular septum of the heart, in the fourth dorsal vertebral body, and in the fourth segment of the liver. A liver biopsy revealed metastatic tissue of neuroendocrine carcinoma (CgA+, synaptophysin+, NSE+, CD56+).

As first-line treatment, a pancreaticoduodenectomy (Whipple procedure) was performed with a concomitant wedge resection and radio-frequency tissue ablation of liver metastases, cholecystectomy, and right hemicolectomy with regional lymphadenectomy.

**Figure 1.**
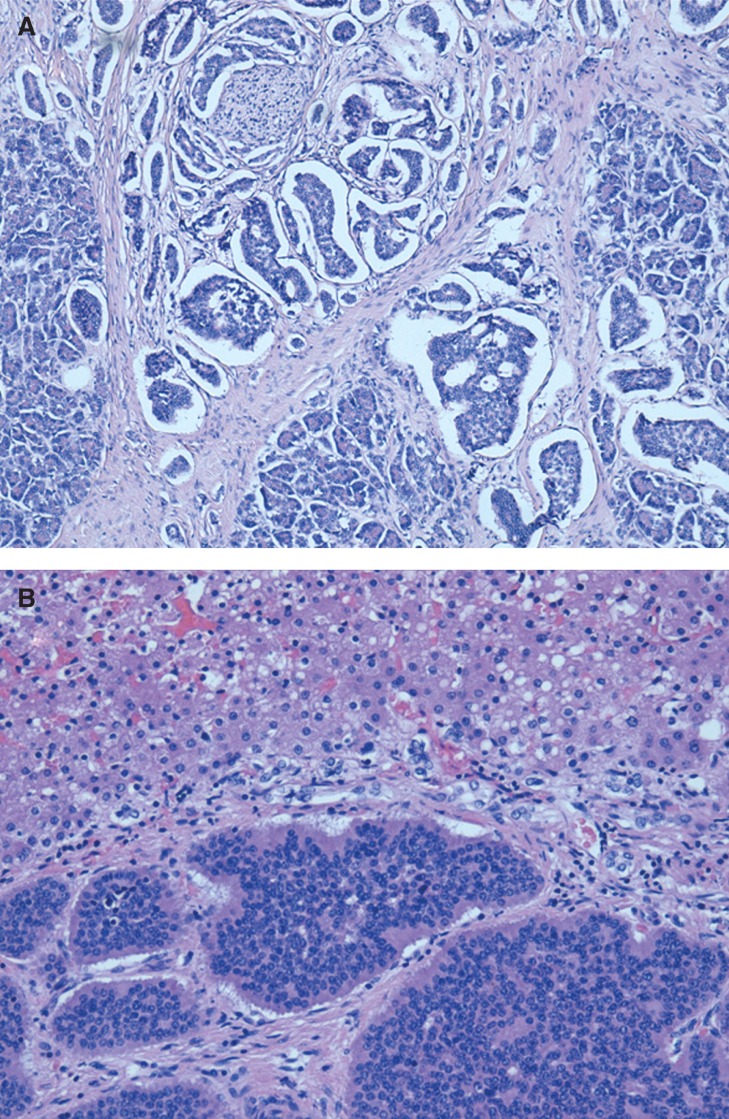
Well-differentiated neuroendocrine carcinoma of the pancreas (A), with liver metastases (B) (original magnification 400×).

The histological and immunohistochemical analyses revealed a well-differentiated neuroendocrine carcinoma involving ileum and pancreas (Figure 1A), immunoreactive to CgA, synaptophysin, and CD56. Immunoreaction for Ki-67 was present in 2% of tumoral cells with rare mitosis. Liver metastases (Figure 1B) and regional lymph node involvement were present. According to the TNM staging, the disease was classified as stage IV carcinoma (AJ10: pT3, N1, M1) (4).

After surgery, the patient received biotherapy with a somatostatin analogue (Octreotide LAR 20 mg), 1 fL i.m. every 28 days and five cycles of peptide receptor-targeted radionuclide therapy with 177Lu-DOTATATE (cumulative dose 560 mCi).

**Figure 2.**
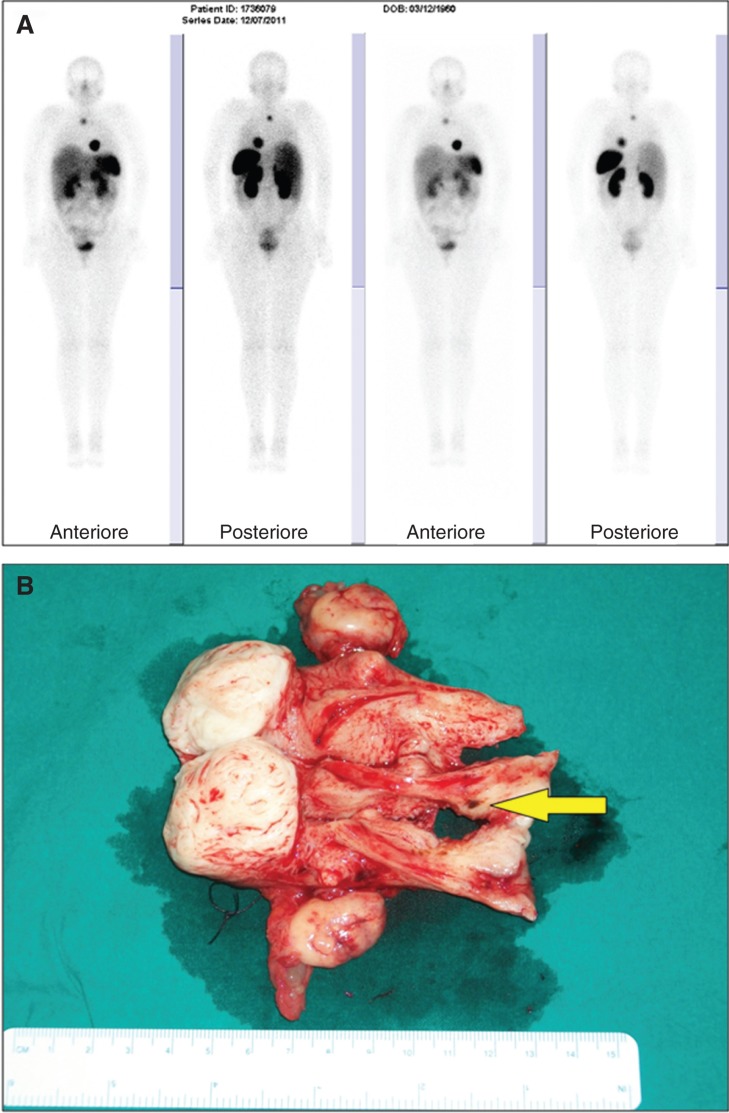
PET scan performed at the end of the fourth cycle of peptide receptor-targeted radionuclide therapy with 177Lu-DOTATATE (A). Gross section of surgical specimens with a circular dark area in the endocervix (yellow arrow) (B).

As follow-up care, the patient underwent several clinical examinations including blood examinations and regular PET scans, which were negative (Figure 2A).

Serum CgA, NSE, serotonin, and urine 5-HIAA were examined at 3, 6, and 9 months after surgery. Their levels became lower and progressively normalized. Serum levels of gastro-intestinal hormones such as somatostatin, gastrin, glucagon, insulin, VIP, and PP remained within normal ranges.

**Figure 3.**
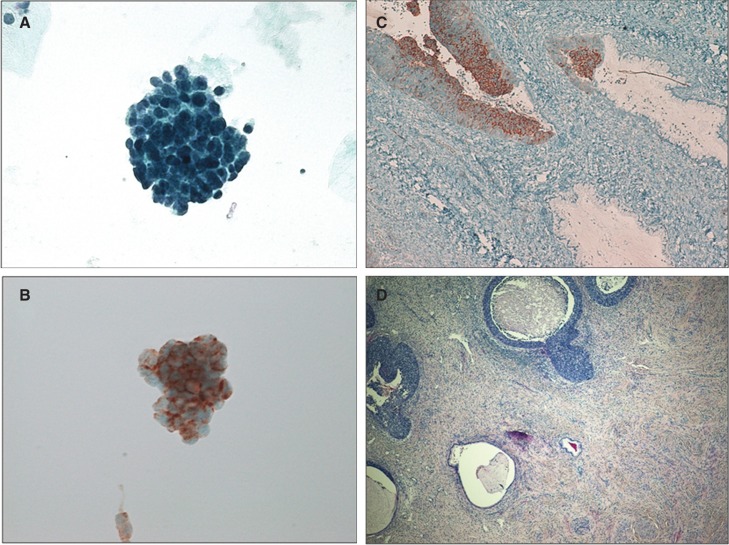
Liquid-based cervical cytology demonstrating atypical cellular clusters (A, B) positive for chromogranin A (B) (original magnification 400×). Histological preparation of the cervix revealing poorly differentiated small cell neuroendocrine tumor involving the cervical crypts (C, D), positive for chromogranin A (C) (original magnification 400×).

One year after the first diagnosis of NET the patient experienced several episodes of abnormal vaginal bleeding. Transvaginal ultrasound (TVS), liquid-based Papanicolaou (pap) test, and hysteroscopy, followed by liquid-based endometrial cytology and biopsy, were performed at the gynecologic department of our institution. TVS revealed a 5 cm intramural myoma at the fundus of the uterus. Cytological and histological samples demonstrated malignant cells that were positive at immunohistochemistry for CgA, synaptophysin, and NSE (Figure 3A and B).

After a multidisciplinary evaluation the patient was submitted to a type A total abdominal hysterectomy and bilateral salpingo-oophorectomy, according to the Morrow and Querleu classification (6).

The gross analysis of the specimens revealed a 45 mm fundal fibroid and a dark-colored circular area of 3 mm maximum size in the endocervix (Figure 2B). No gross abnormality was identified in the fallopian tubes and ovaries. Definitive histological examination showed features of primary cancer in the uterine cervix (Figure 3C and D). The tumor was mainly *in situ* with involvement of the cervical crypts and focally microinvasive. Immunohistochemistry was positive for synaptophysin, CgA, CD56, and NSE, and negative for p63; there were up to 12 typical and atypical mitoses per high-power field (HPF) with almost 100% Ki-67 immunoreactivity. Therefore it was classified as primary poorly differentiated small-cell neuroendocrine cervical carcinoma (SCNCC).

The patient is now under regular follow-up with no signs of active disease.

## Discussion

The diagnosis of NETs is multimodal, based on clinical symptoms, hormone levels, radiological and nuclear imaging, and histological confirmation. Imaging techniques can be initially used to determine the site of primary tumor and to stage the disease. The choice of the imaging technique depends on whether the need is to detect disease in a patient with a suspected NET or to assess the extent of disease in a known case. In the presented case, the first staging procedure performed was a SRS. The SRS demonstrated the presence of a primary entero-pancreatic cancer and three metastases located in the liver, heart, and bone. In the follow-up period, the patient underwent several PET scans with the tracer fluorine-18 (F-18) fluorodeoxyglucose (FDG), all of which were negative for any other primary neuroendocrine lesions. The clinical manifestation of abnormal vaginal bleeding led to a diagnosis of a neuroendocrine uterine malignancy through an endometrial biopsy and a pap smear.

According to the Surveillance, Epidemiology and End Results (SEER) cancer register classification (7), this patient was diagnosed to have multiple primary cancers. More than 80% of multiple primary cancers reported in the SEER database (7) arose in separate or independent organ systems. Pooled data indicate that NETs are associated with other primary intestinal and extra-intestinal non-neuroendocrine malignancies in about 17% of cases and that this is more often seen with primary ileal NETs (29%–52%). The second primary tumor (SPT) site is genito-urinary in 9%–22% of cases (8).

The presented case represents the unusual situation of two primary cancers, occurring at distant sites, having different histological origins even if being both part of the same neuroendocrine group. This is the first case described of neuroendocrine SPT developed in uterine cervix after a GEP-NET primary malignancy. Although both tumors fall under the neuroendocrine group classification, they were not considered to be either multicentric or multifocal tumors because one of them was a small-cell carcinoma and the other was a well-differentiated carcinoma.

Concerning etiological and pathogenetic considerations, it has been speculated that the secretory products of NETs can themselves influence the initiation and promotion of the associated SPT (9,10), but data supporting such affirmation are still lacking. In this case circulating levels of serum CgA, NSE, serotonin, and urine 5-HIAA were initially increased, whereas gastro-intestinal hormones were within normal ranges.

The present case underlines the importance of clinical follow-up after a diagnosis of an entero-pancreatic NET. In case of new symptoms arising during follow-up further investigations must be considered. Female patients, after the diagnosis of GEP-NET, must be recommended to continue screening pap test examinations, for the likelihood of classical squamous or glandular tumor cells and also for neuroendocrine tumor cells. Two tumors of the same group in one patient deserve a search for a reciprocal association that might suggest the presence of an underlying risk factor predisposing to multiple cancers. Genetic predisposition could be a field of future research in these patients.
